# Influence of Different Shaping Techniques on the Aroma Quality and Volatile Metabolites of Green Tea Revealed by Gas Chromatography Electronic Nose and Gas Chromatography–Tandem Mass Spectrometry

**DOI:** 10.3390/foods14050816

**Published:** 2025-02-27

**Authors:** Jiahao Tang, Jiajing Hu, Xianxiu Zhou, Qiwei Wang, Yongwen Jiang, Haibo Yuan, Yujie Wang, Yanqin Yang

**Affiliations:** 1 State Key Laboratory of Tea Plant Biology and Utilization, Anhui Agricultural University, Hefei 230036, China; 2 National Key Laboratory for Tea Plant Germplasm Innovation and Resource Utilization, Tea Research Institute, Chinese Academy of Agricultural Sciences, Hangzhou 310008, China

**Keywords:** green tea, shaping technique, volatile metabolites, GC-E-Nose, GC-MS/MS

## Abstract

The shaping process is recognized as a crucial step in the manufacturing of green tea. However, its influence on aroma quality remains unclear. In this study, the effects of four shaping techniques, including flat green tea (FGT), straight green tea (SGT), phoenix green tea (PGT), and curled green tea (CGT), on the aroma quality and volatile metabolites of green tea were investigated by gas chromatography electronic nose (GC-E-Nose) and gas chromatography–tandem mass spectrometry (GC-MS/MS). The findings indicated that distinct shaping processes significantly influenced the development of the aroma quality and aroma components of green tea. The PGT processing facilitated the attainment of superior aroma quality of green tea. In total, 60 volatile components were identified by GC-MS/MS, with 54 of these compounds being consistently detected across four different shaping techniques. In particular, the PGT processing method was effective in yielding elevated levels of alcohols, esters and ketones. Moreover, 20 key odorants were screened out, with (*E*,*E*)-2,4-decadienal, (*E*,*E*)-2,4-nonadienal, phenylethyl alcohol, and benzeneacetaldehyde proven to be substantial contributors to the overall aromas of green tea under diverse shaping procedures. These key odorants were primarily derived from lipid degradation and the Maillard reaction. GC-E-Nose served as a significant adjunct to sensory evaluation, enabling the swift differentiation of green tea samples that have undergone various shaping processes. These findings offer both theoretical and technical perspectives that may guide the creation of innovative green tea products distinguished by their unique shapes.

## 1. Introduction

Tea is globally acknowledged as one of the three most renowned non-alcoholic beverages, celebrated universally amongst people from all over the world [1,2,3]. Green tea, holding a preeminent position in China’s traditional six major categories, is the most abundant type in China [4,5]. Green tea possesses distinct characteristics such as a high aroma, a mellow taste, and an appealing appearance [6,7]. The processing procedure typically involves the stages of fixation, rolling (or shaping), and drying [8,9]. Currently, investigations into green tea processing technology predominantly concentrate on the fixation and drying stages. For example, the aroma undergoes a progressive transformation, evolving from a faint scent to a chestnut-like scent, and ultimately to a high-fire aroma as the drying temperature increases [10]. Shaping is a crucial step in the manufacturing of premium green tea. This procedure employs either a manual or mechanical external force to guide the tea leaves into corresponding shapes and concurrently dry the tea under specific temperature conditions [11]. Currently, there are scarce reports on the shaping technology of green tea. Different shaping technologies and parameters significantly influence both the external shape and internal quality of tea. Therefore, it is important to clarify the influence of shaping technology on the volatile substances of green tea, which is conducive to the improvement of the processing technology of green tea.

Aroma plays a pivotal role in determining tea flavor, exerting significant influence on its quality [12,13]. It is closely linked to the superiority or inferiority of tea quality and influences consumer preferences. The essence of tea aroma is a complex blend of numerous volatile components in varying concentrations, primarily derived from the transformations of precursor substances such as glycosides, carotenoids, fatty acids, and amino acids during processing [10]. Up to now, more than 700 volatile substances have been isolated and identified from tea [14]. Nevertheless, not all volatile substances contribute directly to the formation of tea aroma due to the distinctive odor characteristics and odor thresholds of various compounds. Only a portion of odor substances with high aroma intensities or high aroma activity values play a vital role in tea aroma quality [15,16].

Advanced instrumental analysis techniques have progressively emerged as indispensable tools for the evaluation of tea aroma [17,18,19,20]. These techniques are characterized by a minimal sampling volume, high sensitivity, excellent repeatability, rapid analytical speed, and high degree of automation [21]. They facilitate the separation and identification of targets from complex mixtures and the quantification of trace and ultra-trace substances. The predominant analytical technology for tea aroma substances primarily encompasses gas chromatography–mass spectrometry (GC-MS), nuclear magnetic resonance, and gas chromatography–tandem mass spectrometry (GC-MS/MS) [22,23,24,25,26,27]. Additionally, it also includes analytical technologies based on sensors such as electronic nose [28,29,30]. Given that tea contains numerous trace or ultra-trace aroma components, it is challenging to achieve the comprehensive characterization of tea aroma components with a single analysis method.

In this study, the influence of different shaping techniques on the aroma quality and volatile metabolites of green tea was comprehensively evaluated through the integration of gas chromatography electronic nose (GC-E-Nose) and GC-MS/MS. The results are expected to provide a theoretical and technical foundation for the processing of premium green tea.

## 2. Materials and Methods

### 2.1. Sample Preparation

**Figure 1:**
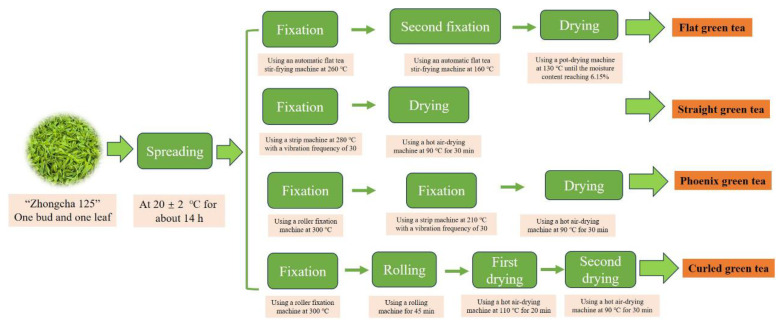
Flowcharts of the manufacturing processes of green tea with different shaping techniques. Independent batches (*n* = 3) were performed.

Freshly harvested tea leaves from Shengzhou City in Zhejiang Province in April 2024 were processed into four distinct types of green tea: flat green tea (FGT), straight green tea (SGT), phoenix green tea (PGT), and curled green tea (CGT). For example, FGT is characterized by its flat appearance, with Xihu Longjing being exemplar of this type, whereas PGT is noted for its resemblance to a phoenix tail, with Anji white tea serving as its representative. The comprehensive manufacturing procedure and specific parameters for the production of FGT, SGT, PGT, and CGT are detailed as follows: the freshly plucked tea leaves from the *Camellia sinensis* cultivar ‘Zhongcha 125’, comprising a single bud and a single leaf, were spread at a temperature of 20 ± 2 °C for a duration of about 14 h. Upon completion of the spreading process, the tea leaves were divided into four equal portions to facilitate the preparation of the four distinct shapes of green tea. A simplified overview of the processing steps is illustrated in Figure 1. For FGT, the spread tea leaves underwent fixation using an automatic flat tea stir-frying machine (Zhejiang Hengfeng technology development Co., Ltd., Shaoxing, China) at 260 °C, followed by a second fixation at 160 °C, and they were subsequently dried using a pot-drying machine (6CH-3.0A, Zhejiang Hengfeng technology development Co., Ltd.) at 130 °C until the moisture content reached 6.15%. For SGT, the spread tea leaves were subjected to fixation using a strip machine (6CL-80-12D, Zhejiang red five ring tea equipment Co., Ltd., Quzhou, China) at 280 °C with a vibration frequency of 30 and then dried in a hot air-drying machine (6CH-6, Zhejiang red five ring tea equipment Co., Ltd.) at 90 °C for 30 min. For PGT, the spread tea leaves were subjected to fixation using a roller fixation machine (6CST-50, Zhejiang red five ring tea equipment Co., Ltd.) at 300 °C, followed by treatment in a strip machine at 210 °C with a vibration frequency of 30, and they were then dried in a hot air-drying machine at 90 °C for 30 min. For CGT, the spread tea leaves underwent fixation in a roller fixation machine at 300 °C. This was succeeded by a rolling process in a rolling machine (6CR-30, Zhejiang red five ring tea equipment Co., Ltd.) for 45 min. Subsequently, the leaves underwent a first round of drying utilizing a hot air-drying machine at 110 °C for 20 min, followed by a secondary drying phase at 90 °C for 30 min. The final tea samples, produced through various shaping techniques, were stored at −20 °C prior to further analysis.

### 2.2. Materials and Reagents

The 20 mL headspace vials and magnetic PTFE/silicone caps were purchased from Agilent Technologies Inc. (Palo Alto, CA, USA). Ethyl decanoate was purchased from TCI Chemical Industry Development Co., Ltd. (Shanghai, China). A blend of *n*-alkanes spanning C7 to C40 was obtained from Sigma-Aldrich (Shanghai, China), and purified water was obtained from Hangzhou Wahaha Group Co., Ltd. (Hangzhou, China). Detailed information regarding the standards of volatile compounds is presented in Table S1.

### 2.3. Sensory Evaluation

The aroma quality of four distinct green tea samples was evaluated by a panel consisting of six specialists with extensive experience, adhering to the Chinese national standard (GB/T 23776-2018) [31]. The specialists consensually agreed to participate and use their data, and ethical permission was not required. Specifically, 3 g of tea samples were introduced into an evaluation cup and infused with 150 mL of boiling water for 4 min. Subsequently, the tea infusion was filtered and transferred into an evaluation bowl for aroma assessment. The aroma types, intensity, and persistence were confirmed through hot sniffing, warm sniffing, and cold sniffing. All 6 participants reached a consensus on the evaluation of aroma attributes. The aroma quality score was calculated using a hundred-point system.

### 2.4. GC-MS/MS Analysis

The headspace solid-phase microextraction technique, adapted from earlier studies [15,32], was employed for the extraction of samples, which underwent various shaping techniques. The manually controlled sampling handle and divinylbenzene/carboxen/polydimethylsiloxane fiber (DVB/CAR/PDMS, 50/30 μm, 2 cm) obtained from Supelco (Bellefonte, PA, USA) were utilized in this experiment. Specifically, 0.5 g of the tea samples and 5 mL of purified water were placed into a 20 mL headspace vial. Following this, the DVB/CAR/PDMS fiber was inserted into the vial prior to incubation at 60 °C for one hour. Finally, the fiber was directed towards the injection port at a temperature of 250 °C, where it underwent five-minute thermal desorption.

A 7890B gas chromatograph system, integrated with a 7000C series triple-quadrupole detector (both manufactured by Agilent Technologies, located in Palo Alto, CA, USA), was employed for analyzing the volatiles, featuring a DB-5MS column (30 m × 0.25 mm × 0.25 μm). Initially, the column’s operational temperature was initiated at 40 °C for a duration of 5 min, progressively increasing to 160 °C at 4 °C/min, maintaining this elevated temperature for another 2 min, and culminating in a final increase to 270 °C at a rate of 10 °C/min (maintained for 2 min). Helium was selected as the carrier gas, flowing at a rate of 1.0 mL/min. The splitless mode was adopted for analysis, with the temperatures of the ion source and transfer line being maintained consistently at 230 °C and 270 °C, respectively. Mass spectrometric analysis was conducted in electron ionization mode at 70 eV, with the mass scanning range spanning from 40 *m*/*z* to 450 *m*/*z*.

The retention index (RI) was calculated by employing normal alkanes from C7 to C40, which served as a basis for comparison with theoretical values. The volatile metabolites were qualitatively evaluated based on a comparison between their distinct mass spectra against those of the standardized references and NIST11 library. Additionally, the RIs were referenced from the databases available at https://webbook.nist.gov/chemistry/ and https://www.flavornet.org/flavornet.html (accessed on 11 September 2024). The accurate quantification of the volatile compounds was achieved via the external standard approach, with the corresponding quantitative information presented in Table S2. For the aroma compounds that lack commercially available standards, quantitative analysis was carried out using the internal standard method with ethyl decanoate.

### 2.5. GC-E-Nose Analysis

A GC-E-Nose instrument (Alpha M.O.S., Toulouse, France) was utilized for the detailed analysis of tea samples subjected to diverse shaping techniques, with replicate analyses performed thrice. The adopted parameters were acquired from the earlier reported literature [32]. Typically, 0.5 g of the tea samples was precisely measured into a 20 mL headspace vial before being exposed to 60 °C for a duration of 20 min. Then, 5000 μL of headspace gas underwent injection into the GC-E-Nose injection port, operated at 200 °C. Volatile substances were adsorbed for 27 s via the utilization of an odor concentration trap (Tenax TA) maintained at 20 °C. The temperature profiles of the MXT-5 column and MXT-1701 column, exhibiting distinct polarity, were as follows: starting from 50 °C (holding for 5 s), ascending progressively to 80 °C at 0.1 °C/s, followed by a sharp transition to 250 °C at 0.4 °C/s (holding for 10 s). Both flame ionization detectors (FIDs) were set to operate at 260 °C.

### 2.6. Odor Activity Value Analysis

The contribution of volatile compounds to the overall aroma was assessed using the odor activity value (OAV) method. Compounds featuring an OAV exceeding one are recognized as vital contributors to the aroma profile of the analyzed samples [15,33]. The mathematical equation employed for this analysis is OAV = C/OT, wherein C (μg/L) denotes the concentration level of each volatile compound, and OT indicates its odor threshold in water.

### 2.7. Statistical Analysis

Three independent batches of green tea processing, comprising both experimental and validation batches, were conducted in accordance with the same methodology. Partial least squares discrimination analysis (PLS-DA) was implemented via the application of SIMCA-P 13.0 (Umetrics, Umea, Sweden). The peak areas obtained from GC-E-Nose and the contents of volatile compounds obtained from GC-MS/MS were designated as the independent variables, respectively, whereas the results of the sensory evaluation were defined as the dependent variables. Significance analysis was evaluated on the contents of volatile compounds in green tea samples by means of one-way analysis of variance (ANOVA) using SPSS 20.0 (Chicago, IL, USA), followed by Duncan’s test. Venn diagrams were constructed by employing the robust Venny software (https://jvenn.toulouse.inrae.fr/app/example.html (accessed on 3 September 2024)). Bar graphs were constructed using Origin software 2024 (Northampton, MA, USA). Hierarchical cluster analysis (HCL) was performed on 21 significant differential volatile compounds utilizing Metware Cloud (https://cloud.metware.cn (accessed on 20 September 2024)), adhering to the criteria of *p* < 0.05 and VIP > 1. Red denotes higher than the average concentration, while green signifies lower than the average concentration. The darker the color, the higher the concentration.

## 3. Results and Discussion

### 3.1. Analysis of the Effects of Different Shaping Techniques on the Aroma Quality of Green Tea by Sensory Evaluation

In this study, green tea samples obtained from four distinctive shaping methods, namely PGT, FGT, CGT, and SGT, were subjected to sensory evaluation. The results indicated that significant variations in the overall aroma quality of green tea were observed under diverse shaping procedures (Table S3). Specifically, the PGT sample attained the highest score of 89.6, manifesting a delightful chestnut-like aroma. The FGT sample exhibited a cooked chestnut-like aroma, attaining a score of 88.17. The CGT presented a fresh scent, scoring 87.33, whereas the SGT exhibited a neutral tea aroma, earning a score of 86.4. In summary, the PGT processing proved beneficial to obtain high-quality aroma of green tea. It is possible that during the production process of PGT, various high-temperature steps (such as fixation and drying) were performed with multiple types of tea-manufacturing apparatus (such as a roller fixation machine, strip machine, and hot air-drying machine) to prompt the occurrence of lipid oxidation degradation and the Maillard reaction, thereby enhancing the aroma quality [2,16].

### 3.2. Analysis of the Effects of Different Shaping Techniques on the Volatile Profiles of Green Tea Using GC-E-Nose

Traditional sensor-based electronic noses face challenges such as sensor instability and odor contamination, which reduce the response values. As an innovative electronic nose technology, GC-E-Nose integrates two gas chromatography columns with disparate polarities to efficiently analyze the volatile profiles of target samples. With the aid of advanced algorithm models such as PLS-DA incorporated within GC-E-Nose, it allows for the rapid differentiation of samples with diverse odor characteristics. In this study, the volatile profiles of green tea subjected to diverse shaping methods were analyzed by GC-E-Nose. As illustrated in Figure S1, the volatile profiles collected from the MXT-5 and MXT-1701 columns with different shaping methods exhibited a distinct disparity.

**Figure 2:**
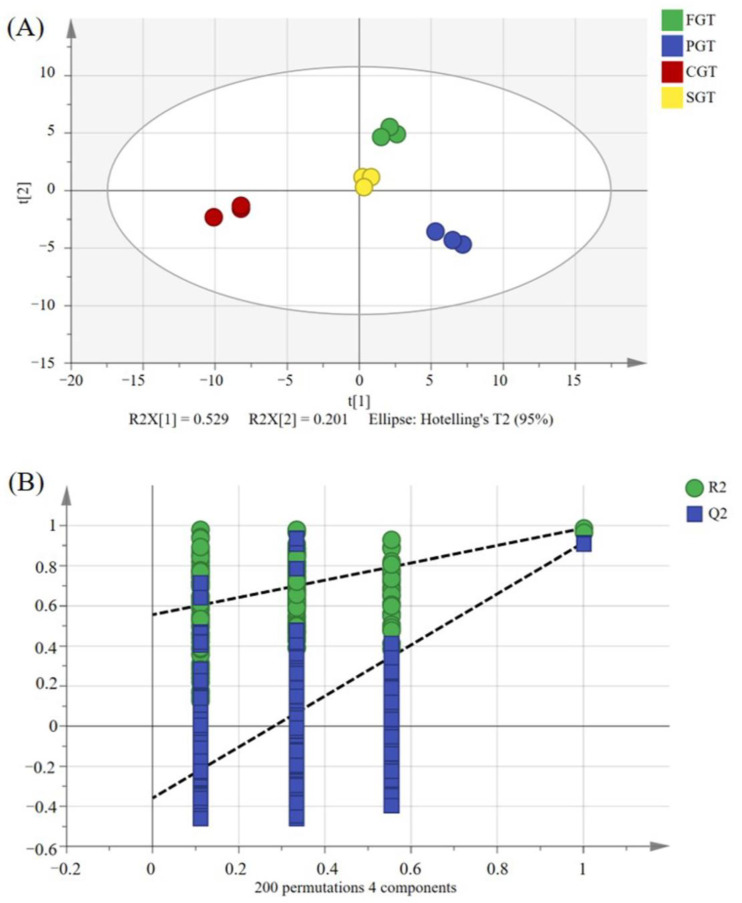
PLS-DA performed on the volatile profiles in tea samples with four distinct shaping techniques obtained by GC-E-nose. (**A**) Score plots of PLS-DA (R^2^Y = 0.987, Q^2^ = 0.909); (**B**) permutation test conducted 200 times with R^2^ = (0.0, 0.561) and Q^2^ = (0.0, −0.375). FGT represents flat green tea. PGT represents phoenix green tea. CGT represents curled green tea. SGT represents straight green tea.

To further investigate the distinctions in volatile profiles among tea samples subjected to different shaping methods, a supervised PLS-DA model was employed for analysis. As shown in Figure 2A, a distinct discrimination was achieved in the score plots of PLS-DA, indicating that there were substantial differences in their aroma profiles. The model’s parameters indicated an R^2^Y of 0.987, with a Q^2^ of 0.909. Generally, the closer the R^2^Y value is to 1, the stronger the explanatory power that the model possesses. Moreover, a higher Q^2^ value indicates better predictive accuracy. Based on the aforementioned results, the model exhibited robust explanatory and predictive capabilities. To further verify the reliability of the model, 200 iterations of permutation testing were conducted. The results for R^2^ = (0.0, 0.561) and Q^2^ = (0.0, −0.375) demonstrated that the model was reliable and not overfitted (Figure 2B). Variable importance in projection (VIP) could quantify the contribution of each volatile compound to the classification ability of the model. Typically, the higher the VIP value of a volatile compound, the greater its contribution to the classification. In this study, a total of 23 variables with VIP greater than 1 were screened out (Figure S2). In conclusion, the GC-E-Nose functioned as an important complement to the sensory evaluation, and could rapidly differentiate tea samples obtained from diverse processing methods.

### 3.3. Analysis of the Effects of Different Shaping Techniques on the Volatile Metabolites of Green Tea Using GC-MS/MS

#### 3.3.1. Comparative Analysis of Volatile Metabolites

**Figure 3:**
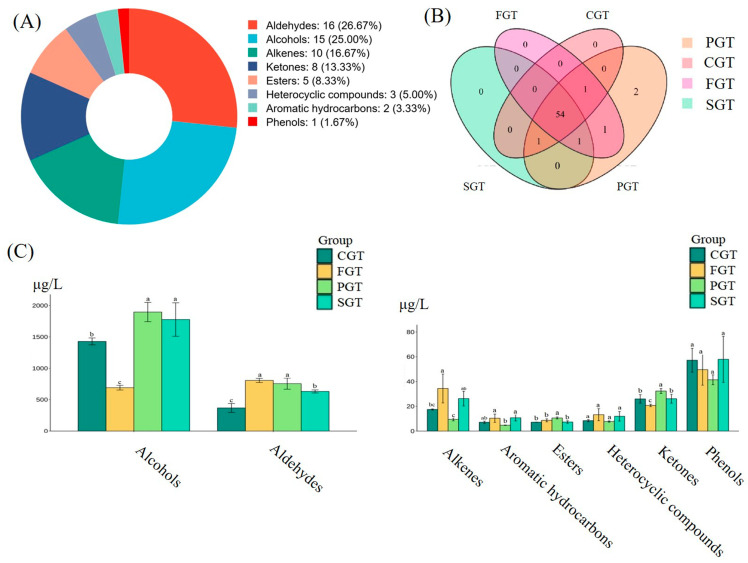
Volatile compounds in tea samples with four distinct shaping techniques analyzed by GC-MS/MS. (**A**) Pie chart of the proportion of various compounds; (**B**) Venn diagrams of volatile compounds; (**C**) Comparison of different categories of volatile compounds. Different letters indicate significant differences (*p* < 0.05). FGT represents flat green tea. PGT represents phoenix green tea. CGT represents curled green tea. SGT represents straight green tea.

Clarifying the effect of diverse shaping techniques on volatile components will facilitate the improvement of the manufacturing process of green tea. In this study, a total of 60 volatile compounds were identified, as detailed in Table S4. These volatile compounds primarily comprised 16 aldehydes, 15 alcohols, 10 alkenes, 8 ketones, 5 esters, 3 heterocyclic compounds, 2 aromatic hydrocarbons, and 1 phenol, with aldehydes (26.67%) and alcohols (25.00%) representing the primary aroma categories (Figure 3A). Moreover, 54 common volatile compounds were detected across four shaping techniques (Figure 3B). Notably, 1-heptanol and citral were exclusively detected in the PGT, while 1-nonanal was solely detected in the FGT and PGT. Additionally, *cis*-2-penten-1-ol was detected in the FGT, SGT, and PGT, and acetic acid, butyl ester was present in all tea samples except for the SGT. Nerol was detected in all samples except for the FGT.

Distinct shaping procedures exert a considerable influence on the contents of volatile compounds in green teas. As illustrated in Figure 3C, the contents of various categories of volatile compounds exhibited obvious differences under distinct shaping processes. Notably, alcohols attained the highest contents among the aroma components, endowing green tea with floral and fruity fragrances. Prior studies indicate that alcohols were the predominant compounds produced under various light irradiation spreading conditions, aligning with our findings [32]. Specifically, the content of alcohols was the highest under the PGT, reaching 1895.89 μg/L, whereas it was the lowest under the FGT, merely 691.21 μg/L. The variations in heat treatment processes significantly influence the Maillard reaction and the oxidative degradation of lipids, which in turn affects the production of alcohols during various shaping processes. For instance, phenylethyl alcohol, a notable alcohol compound, is produced through the thermal degradation of phenylalanine, whereas 1-octen-3-ol is generated from the oxidative degradation of the unsaturated fatty acid linoleic acid [2]. Aldehydes play an important role in the formation of green tea aroma. The content of aldehydes was the maximum in the FGT (806.68 μg/L) and the minimum in the CGT (368.11 μg/L). Olefins typically exhibit distinctive aromatic odors, possessing the highest content in the FGT (34.40 μg/L). The contents of olefin compounds for other shaping methods were ordered as follows: SGT (26.26 μg/L) > CGT (17.47 μg/L) > PGT (9.45 μg/L). The content of ketones was the highest in the PGT (32.33 μg/L) and the lowest in the FGT (20.72 μg/L), with minimal disparity between the CGT and SGT samples. The content of heterocyclic substances reached the highest level in the FGT (13.24 μg/L) and the lowest in the PGT (7.71 μg/L). The levels of aromatic hydrocarbons in the SGT (10.76 μg/L) and FGT (10.39 μg/L) were not significantly different, with the contents in CGT and PGT being 7.13 μg/L and 4.73 μg/L, respectively. The contents of esters under the four shaping methods were as follows: PGT (10.58 μg/L), FGT (8.68 μg/L), SGT (7.33 μg/L), and CGT (7.18 μg/L). The content of phenols reached the highest level in the SGT (57.96 μg/L) and the lowest in the PGT (41.40 μg/L). The aforementioned results underscored that different shaping procedures have a substantial impact on the number and contents of the aroma components of green tea.

#### 3.3.2. Multivariate Statistical Analysis

**Figure 4:**
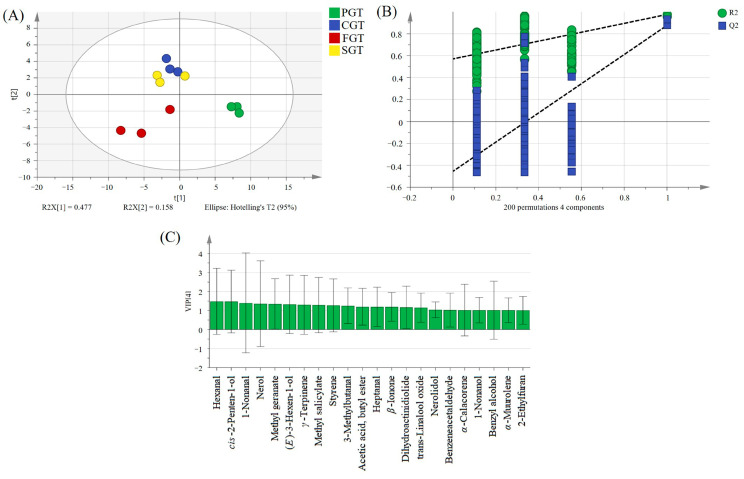
PLS-DA performed on the volatile compounds in tea samples with four distinct shaping techniques obtained from GC-MS/MS. (**A**) Score plot of PLS-DA (R^2^Y = 0.971, Q^2^ = 0.818); (**B**) permutation test conducted 200 times with R^2^ = (0.0, 0.561) and Q^2^ = (0.0, −0.496); (**C**) a total of 22 key differential volatile substances with VIP greater than 1. FGT represents flat green tea. PGT represents phoenix green tea. CGT represents curled green tea. SGT represents straight green tea.

**Figure 5:**
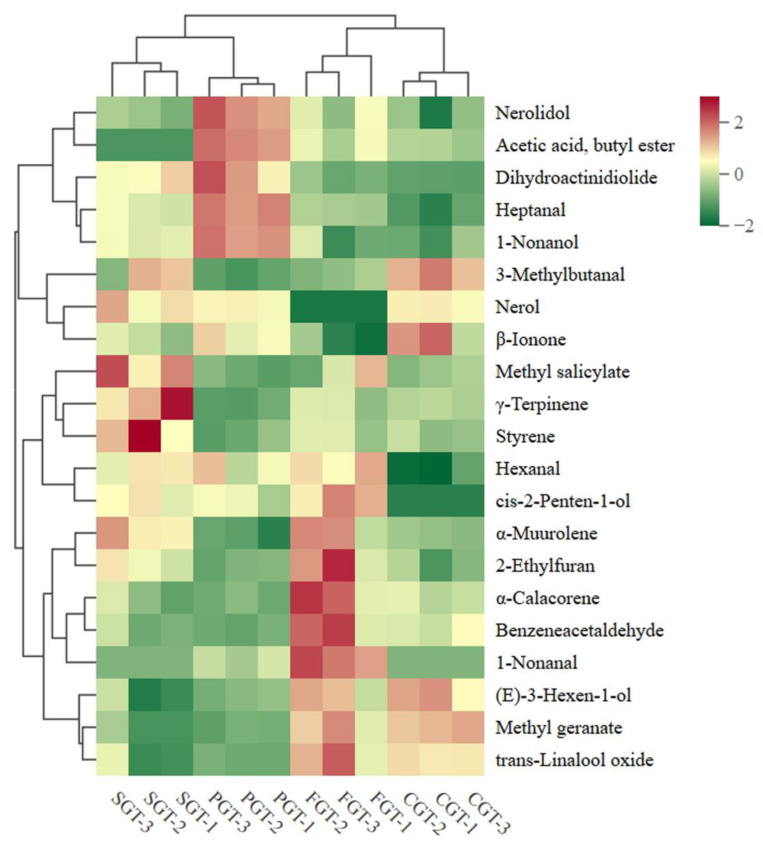
Hierarchical cluster analysis of 21 volatile compounds in tea samples with four distinct shaping techniques according to *p* < 0.05 and VIP > 1. FGT represents flat green tea. PGT represents phoenix green tea. CGT represents curled green tea. SGT represents straight green tea.

To investigate the key differential components in tea samples under different shaping methods, PLS-DA was conducted. As depicted in Figure 4A, excellent discrimination was achieved. The PGT samples were located in the fourth quadrant of the score plots, whereas the CGT and SGT were distributed in the second quadrant. The FGT samples were mainly positioned in the third quadrant. The parameters of the PLS-DA model are presented as follows: the cumulative R^2^Y = 0.971 and Q^2^ = 0.818, indicating that the model displayed commendable explanatory and predictive capabilities. To further verify the stability of the model, 200 iterations of permutation testing were conducted. The obtained parameters were R^2^ = (0.0, 0.561) and Q^2^ = (0.0, −0.496) (Figure 4B), demonstrating that the model was dependable and not overfitted. Volatile compounds with VIP values greater than 1 are typically recognized as significant volatile compounds. As illustrated in Figure 4C, a total of 22 volatile compounds with VIP values exceeding 1 were identified. Adhering to the screening principles of *p* < 0.05 and VIP > 1, a total of 21 volatile compounds were selected as pivotal compounds for distinguishing tea samples under different shaping methods, with aldehydes, esters, and alcohols being the predominant volatiles. Furthermore, hierarchical cluster analysis was performed on the 21 key differential volatile compounds. As can be seen from Figure 5, volatiles such as methyl salicylate, *γ*-terpinene, and styrene possessed higher contents in the SGT compared to the other treatment groups. Nerolidol, acetic acid, butyl ester, dihydroactinidiolide, 1-heptanol, and 1-nonanol exhibited higher contents in the PGT than the other treatment groups. 2-Ethylfuran, *α*-calacorene, benzeneacetaldehyde, 1-nonanal, and *α*-muurolene exhibited higher contents in the FGT than the other treatment groups. Representative compounds such as 3-methylbutanal *β*-ionone, (*E*)-3-hexen-1-ol, and methyl geranate displayed higher contents in the CGT than the other treatment groups. Due to the disparity in technological parameters and so forth, diverse shaping processes exhibit distinct thermophysical and chemical effects, thereby inducing considerable variations in the levels of volatile metabolites.

#### 3.3.3. OAV Analysis

**Figure 6:**
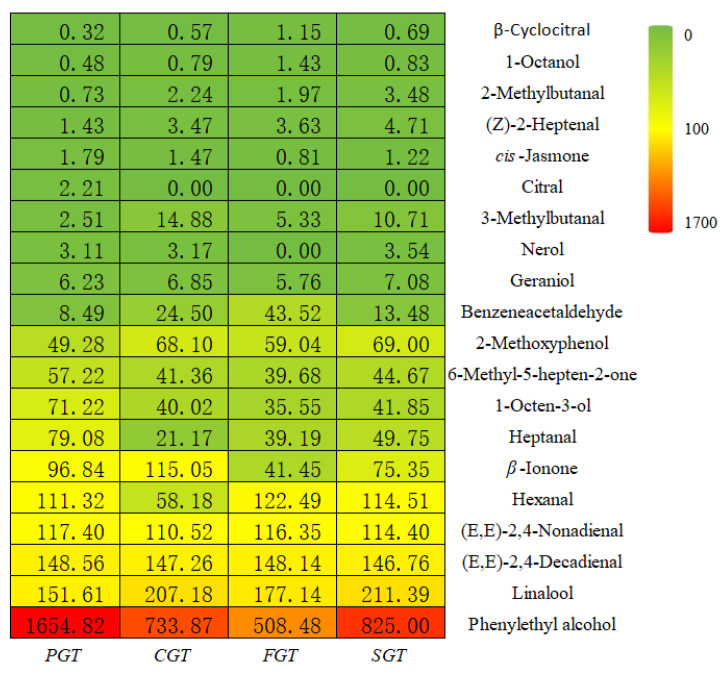
Comparison of 20 key odorants with OAV > 1 in tea samples with four distinct shaping techniques.

Not all volatile compounds necessarily contribute to the fragrance of green tea. Hence, OAV analysis is indispensable to further ascertain those volatile constituents that significantly contribute to the overall aroma of diversely shaped samples. Generally speaking, when the OAV of a volatile compound is greater than 1, it can be deduced that this compound makes an important contribution to the overall aroma. The higher the OAV, the greater its impact on the aroma quality [34]. In this study, the odor thresholds and calculated OAVs of the volatile components of green tea with four different shaping techniques are listed in Table S5. A total of 20 volatile compounds with OAVs greater than 1 were screened out from the tea samples produced via the four shaping methods (Figure 6). Based on the magnitude of the OAVs, these compounds were primarily divided into two distinct groups. The first group encompassed volatiles, including 3-methylbutanal, 2-methylbutanal, heptanal, 1-octen-3-ol, 6-methyl-5-hepten-2-one, benzeneacetaldehyde, 1-octanol, 2-methoxyphenol, *β*-cyclocitral, nerol, geraniol, citral, *cis*-jasmone, and (*Z*)-2-heptenal, characterized by OAVs below 100. The second group typically featured volatile compounds such as *β*-ionone, hexanal, (*E*,*E*)-2,4-nonadienal, (*E*,*E*)-2,4-decadienal, linalool, and phenylethyl alcohol, which exhibited OAVs exceeding 100.

Among the volatiles with OAVs surpassing 100, the OAVs of (*E*,*E*)-2,4-nonadienal and (*E*,*E*)-2,4-decadienal displayed minimal fluctuations under the four distinct shaping methods. The OAV of hexanal in the PGT, FGT, and SGT samples was notably higher compared to CGT. *β*-Ionone is a pivotal ketone compound that affects the aroma profile of green tea and typically exhibits a floral fragrance [14]. In this study, its OAV reached the highest value in the CGT (115.05) and the lowest in the FGT (41.45). As a representative monoterpene alcohol in tea, linalool primarily presents citrus and floral scents. Its OAV attained the highest value in the SGT (211.39) and the lowest in the PGT (151.61). Phenylethyl alcohol also presents an appealing fruity and floral fragrance [2]. Its OAV surpassed 500 in all four shaping methods, attaining the maximum in the PGT (1654.82). To sum up, the diverse shaping techniques significantly influenced the OAVs of the aroma compounds, thereby influencing the manifestation of the fragrance style.

### 3.4. Discussion on the Potential Formation Pathways of Key Aroma Components in Green Tea with Different Shaping Techniques

**Figure 7:**
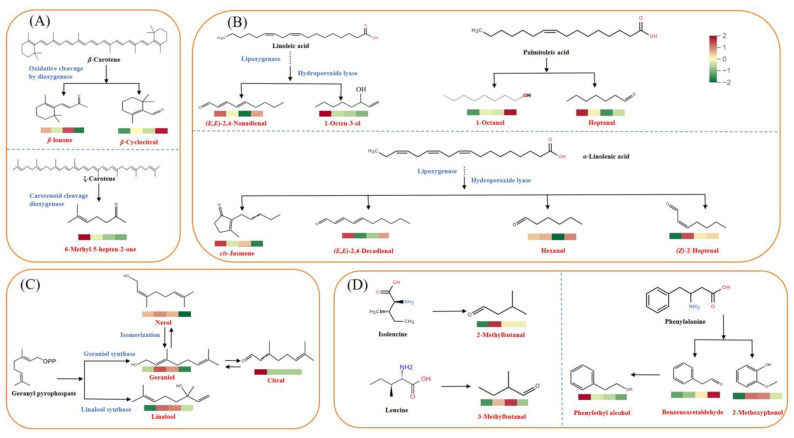
Content variations and key metabolic pathways of important compounds in tea samples with four distinct shaping techniques. (**A**) Carotenoid degradations; (**B**) Lipid oxidation degradations; (**C**) Glycosides as precursors; (**D**) Maillard reaction pathway. Different colored boxes represent the contents of metabolites in PGT, SGT, CGT, and FGT from left to right. Red and green colors indicated metabolite levels above and below the average, respectively.

The volatile compounds present in tea are intricately blended in specific proportions to generate its distinctive aroma. The mechanisms underlying the formation of these volatile compounds are highly complex. Following the metabolic pathways, the aforementioned 20 essential aroma components were primarily divided into four categories: carotenoid degradations (3), lipid oxidation degradations (8), glycosides as precursors (4), and Maillard reaction products (5) (Figure 7). The lipid degradation and Maillard reaction were the primary formation pathways for key aroma components of green teas subjected to four distinct shaping methods.

To be specific, *β*-cyclocitral, *β*-ionone, and 6-methyl-5-hepten-2-one were derived from the oxidative degradation of carotenoids [2]. *β*-Cyclocitral and *β*-ionone originated from the dioxygenase oxidation and cleavage of *β*-carotene. *β*-Cyclocitral exhibited the highest content in the FGT (3.44 μg/L) and the lowest in the PGT (0.95 μg/L) (Figure 7A). *β*-Ionone exhibited the lowest content in the FGT (0.41 μg/L), yet it displayed comparable levels in the PGT (0.97 μg/L) and CGT (1.15 μg/L). 6-Methyl-5-hepten-2-one originated from ξ-carotene, and it exhibited the highest content in the PGT (9.15 μg/L).

Volatiles such as heptanal, hexanal, 1-octen-3-ol,1-octanol, (*Z*)-2-heptenal, (*E*,*E*)-2,4-decadienal, (*E*,*E*)-2,4-nonadienal, and *cis*-jasmone originated from the oxidation degradation of unsaturated fatty acids such as *α*-linolenic acid, linoleic acid, and palmitoleic acid [34,35,36]. As shown in Figure 7B, (*E*,*E*)-2,4-decadienal, *cis*-jasmone, (*Z*)-2-heptenal, and hexanal originated from the precursor of *α*-linolenic acid, with the content of (*E*,*E*)-2,4-decadienal being comparable across the four shaping methods and the content of *cis*-jasmone exhibiting the highest level in the PGT and the lowest level in the FGT. The concentration of (*Z*)-2-heptenal was notably higher in the SGT, FGT, and CGT when compared to that in the PGT. Hexanal exhibited the highest content in the FGT (551.20 μg/L), followed by SGT (515.30 μg/L), PGT (500.94 μg/L), and CGT (261.81 μg/L). Both heptanal and 1-octanol originated from the oxidation degradation of palmitoleic acid. The content of heptanal in the PGT (71.17 μg/L), SGT (44.77 μg/L), and FGT (35.27 μg/L) was significantly higher than that in the CGT (19.05 μg/L), whereas 1-octanol exhibited the maximum content in the FGT (4.29 μg/L) and the minimum in the PGT (1.43 μg/L). The synthetic precursor of 1-octen-3-ol and (*E*,*E*)-2,4-nonadienal was linoleic acid, with the content of 1-octen-3-ol in the PGT (71.22 μg/L) being significantly higher than in the CGT (40.02 μg/L), FGT (35.55 μg/L), and SGT (41.85 μg/L) and the content of (*E*,*E*)-2,4-nonadienal exhibiting the highest level in the PGT (7.04 μg/L). 

Geraniol, linalool, nerol, and citral are glycoside-derived volatiles (Figure 7C). By employing geranyl pyrophosphate as the precursor substrate, geraniol and linalool were synthesized via the catalysis of geraniol synthase and linalool synthase, respectively [2,7]. Geraniol and linalool are prevalent volatile compounds in tea, exerting a pivotal influence on the development of tea aroma [19]. The content of linalool fluctuated slightly across the four shaping methods, around approximately 100 μg/L, whereas the content of geraniol varied around 48 μg/L. In addition, citral was generated by the oxidation of geraniol [37]. It is noteworthy that citral was exclusively identified in the PGT sample. Nerol was isomerized from geraniol and exhibited the highest level among the vital odorants, with its content being around 950 μg/L in the PGT, CGT, and SGT samples.

Benzeneacetaldehyde, phenylethyl alcohol, 3-methylbutanal, 2-methoxyphenol, and 2-methylbutanal were derived from the degradation of amino acids, with benzeneacetaldehyde, phenylethyl alcohol, and 2-methoxyphenol being derived from phenylalanine [2,7,38]. These volatiles play pivotal roles in generating fruity and floral odors. Benzeneacetaldehyde was produced from phenylalanine through Strecker degradation, and its content in the FGT (52.23 μg/L) was markedly higher than that for the other three shaping techniques (Figure 7D). Phenylethyl alcohol displayed the highest content in the PGT (579.19 μg/L), followed by the SGT (288.75 μg/L), CGT (256.86 μg/L), and FGT (177.97 μg/L) samples. 2-Methoxyphenol was a prevalent phenolic compound in tea, and its content showed no significant variations among the four shaping methods. 3-Methylbutanal was derived from the degradation of leucine, and its content was significantly higher in the CGT and SGT samples compared to that in the PGT and FGT samples. 2-Methylbutanal was derived from the degradation of isoleucine and possessed a content comparable with that of 3-methylbutanal in the PGT, FGT, and SGT samples.

## 4. Conclusions

This study investigated the effect of diverse shaping techniques on the aroma quality and volatile metabolites of green tea, employing advanced analytical techniques such as GC-E-Nose and GC-MS/MS. Our findings demonstrated that distinct shaping procedures notably influenced the overall aroma quality. The specific procedure designated as PGT yielded a superior aroma quality, characterized by a pleasant chestnut-like aroma. Through the application of GC-MS/MS, 60 volatile components were identified, of which 21 were selected as key differentiators among tea samples subjected to various shaping techniques, based on VIP > 1.0 and *p* < 0.05. Furthermore, 20 critical odorants with OAVs > 1.0 were pinpointed. Notably, (*E*,*E*)-2,4-decadienal, (*E*,*E*)-2,4-nonadienal, phenylethyl alcohol, and benzeneacetaldehyde were confirmed as substantial contributors responsible for the aroma of green tea. Lipid degradation and Maillard reactions were the primary pathways contributing to the generation of these pivotal odorants under diverse shaping conditions. Additionally, GC-E-Nose served as an effective tool for the rapid differentiation of green tea samples subjected to various shaping methods, complementing sensory evaluation. Future research will involve the use of stable isotope tracer technology to explore the mechanisms underlying the formation of these crucial aroma components across different shaping processes. Moreover, a molecular docking technique will be employed to elucidate the perceptual mechanisms of these vital aroma components at the molecular level. These findings provide a theoretical foundation for enhancing processing technologies related to green tea.

## Data Availability

The original contributions presented in this study are included in the article/Supplementary Materials. Further inquiries can be directed to the corresponding authors.

## Supplementary Materials

The following supporting information can be downloaded at https://www.mdpi.com/article/10.3390/foods14050816/s1: Table S1. The standard information of volatile compounds used in this study; Table S2. The quantification information of the volatile compounds in tea samples with four distinct shaping techniques obtained from GC-MS/MS; Table S3. The aroma evaluation of green tea with different shaping techniques, Table S4. The volatile compounds in tea samples with four distinct shaping techniques obtained from GC-MS/MS; Table S5. The OAVs of volatile compounds in tea samples with four distinct shaping techniques [14,22,39,40]. Figure S1. The volatile fingerprints of green tea with different shaping techniques obtained from the MXT-5 and MXT-1701 columns in parallel; Figure S2. A total of 23 variables with VIP greater than 1obtained from GC-E-Nose.
