# Supplementary material for: Influence of Different Shaping Techniques on the Aroma Quality and Volatile Metabolites of Green Tea Revealed by Gas Chromatography Electronic Nose and Gas Chromatography–Tandem Mass Spectrometry
Source: Foods. 2025 Feb 27;14(5):816. doi: 10.3390/foods14050816 (PMC11898589; doi:10.3390/foods14050816)
Supplement: Supplementary file 1 [file foods-14-00816-s001.zip › foods-3362158-supplementary.pdf]

Supporting Information for

Influence of Different Shaping Techniques on the Aroma Quality and Volatile Metabolites of Green Tea Revealed by Gas Chromatography Electronic Nose and Gas Chromatography–Tandem Mass Spectrometry

Jiahao Tang [1,2], Jiajing Hu [2], Xianxiu Zhou [2], Qiwei Wang [2], Yongwen Jiang [2], Haibo Yuan [2], Yujie Wang [1,*] and Yanqin Yang [2,*]

[1] State Key Laboratory of Tea Plant Biology and Utilization, Anhui Agricultural University, Hefei 230036, China
[2] National Key Laboratory for Tea Plant Germplasm Innovation and Resource Utilization, Tea Research Institute, Chinese Academy of Agricultural Sciences, Hangzhou 310008, China
* Correspondence: wangyj@ahau.edu.cn (Y.W.); yangyq@tricaas.com (Y.Y.)

Table S1 The standard information of volatile compounds used in this study.

Standards	Information			
	Purity	Brand	City	Country
Phenylethyl alcohol	99.5%	J&K	Shanghai	China
Linalool	98%	J&K	Shanghai	China
Acetic acid, butyl ester	>98%	Aladdin	Shanghai	China
1-Octen-3-ol	98%	Aladdin	Shanghai	China
Nerol	>98%	Aladdin	Shanghai	China
Nerolidol	97%	Aladdin	Shanghai	China
2-Methoxyphenol	98%	Aladdin	Shanghai	China
Hexanal	>98%	TCI	Shanghai	China
Heptanal	>98%	TCI	Shanghai	China
1-Nonanal	>95%	TCI	Shanghai	China
β-Ionone	>95%	TCI	Shanghai	China
1-Heptanol	98%	TCI	Shanghai	China
Benzyl alcohol	99.5%	Macklin	Shanghai	China
Benzaldehyde	98%	Macklin	Shanghai	China
(*E,-E*)-2,4-Nonadienal	>90%	Macklin	Shanghai	China
(*E,-E*)-2,4-Decadienal	>90%	Macklin	Shanghai	China
Methyl salicylate	>99%	Macklin	Shanghai	China
(*Z*)-Hexanoic acid, 3-hexenyl ester	98%	Macklin	Shanghai	China
cis-Jasmone	98%	Macklin	Shanghai	China
3-Nonen-2-one	>96%	Macklin	Shanghai	China
Geranyl acetone	97%	Meryer	Shanghai	China

1-Nonanol	99.5%	Yingxin	Shanghai	China
Citral	≥98%	Yingxin	Shanghai	China
cis-2-Penten-1-ol	⩾95%	Macklin	Shanghai	China
6-Methyl-5-hepten-2-one	> 98%	Mreda	Beijing	China
(*E*,-*E*)-2,4-Heptadienal	≥90%	TRC	Toronto	Canada
Indole	>97%	Solarbio	Beijing	China
Geraniol	>98%	Yuanye	Shanghai	China
Dihydroactinidiolide	98%	HARVEYBIO	Beijing	China

Table S2 The quantification information of the volatile compounds in tea samples with four distinct shaping techniques obtained from GC-MS/MS.

RT (min)	Compounds	Standard curves	R^2
3.22	*cis*-2-Penten-1-ol	y=226.627x+6104.328	0.999
6.02	Hexanal	y=340.669x+19923.198	0.995
6.81	Acetic acid, butyl ester	y=15270.332x+202172.846	0.999
10.36	Heptanal	y=9241.721x+92782.270	0.994
12.88	Benzaldehyde	y=25619.633x-15854.492	0.998
13.37	1-Heptanol	y=19815.412x+161373.898	0.997
14.05	1-Octen-3-ol	y=9349.845x+108.198	0.991
14.13	6-Methyl-5-hepten-2-one	y=1323.637x+288.782	0.999
15.27	(*E*,-*E*)-2,4-Heptadienal	y=62342.784x-186221.714	0.999
16.01	Benzyl alcohol	y=15789.314x-208.879	0.995
18.3	2-Methoxyphenol	y=692.472x+1647.855	0.997
19.09	Linalool	y=123650.258x+572854.127	0.999
19.16	1-Nonanal	y=40.614x+2917.349	0.992
19.53	Phenylethyl alcohol	y=1784.805x-19447.349	0.999
20.36	3-Nonen-2-one	y=170.260x-33.412	0.998
22.18	1-Nonanol	y=53658.700x-131828.772	0.999
22.51	Methyl salicylate	y=5616.107x-3850.712	0.998
23.52	(*E*,-*E*)-2,4-Nonadienal	y=75381.831x-428638.068	0.993
24.05	Nerol	y=5134.434x+7587.629	0.998
24.78	Geraniol	y=100350.613x-47135.488	0.999
25.3	Citral	y=9639.067x+56418.992	0.997
25.7	Indole	y=147743.223x-99257.158	0.999
25.71	Dihydroactinidiolide	y=6021.934x+266.670	0.998
27.31	(*E*,-*E*)-2,4-Decadienal	y=186290.348x-728562.053	0.999
29.2	(*Z*)-Hexanoic acid, 3-hexenyl ester	y=26307.948x-12448.430	0.999
29.7	*cis*-Jasmone	y=2742.521x+11721.773	0.999
31.4	Geranyl acetone	y=15771.902x-1529.554	0.999
32.3	*β*-Ionone	y=745.084x+297.534	0.996
33.85	Nerolidol	y=145097.591x-239590.561	0.999

Table S3 The aroma evaluation of green tea with different shaping techniques.

Shaping techniques	Aroma	
	Comments	Scores of overall aroma quality
CGT	Fresh scent	87.33±0.58cd
PGT	Chestnut-like aroma	89.6±0.53a
FGT	Cooked chestnut-like aroma	88.17±0.29b
SGT	Tea aroma	86.4±0.69d

Note: FGT represents flat green tea. PGT represents phoenix green tea. CGT represents curled green tea. SGT represents straight green tea. Different letters indicated a significant difference ($p < 0.05$).

Table S4 The volatile compounds in tea samples with four distinct shaping techniques obtained from GC-MS/MS.

No.	RT[a] (min)	RI[b]	RI[c]	Compounds	Contents (μg/L)				ID	VIP	*P*
					PGT	CGT	FGT	SGT			
1	2.68	707	697	3-Methylbutanal	1.26±0.34c	7.44±0.89a	2.67±0.55bc	5.36±2.68ab	MS，RI	1.25	0.003
2	2.79	710	681	2-Methylbutanal	1.1±0.16b	3.36±1.41bc	2.96±1.89bc	5.22±2.35a	MS，RI	0.98	0.09
3	3.04	716	716	1-Penten-3-ol	1.09±0.23b	2.89±0.15a	2.66±1.38ab	3.14±1.04a	MS，RI	0.78	0.075
4	3.22	720	740	*cis*-2-Penten-1-ol	177.63±43.83b	/	289.49±55.1a	215.12±31.12b	MS, RI, Std	1.48	0
5	3.24	721	715	Pentanal	2.18±0.41a	4.57±1.09a	5.6±2.59a	2.92±1.7a	MS，RI	0.88	0.121
6	3.26	721	717	2-Ethylfuran	1.52±0.19b	1.62±0.53b	3.8±1.27a	2.74±0.38ab	MS，RI	1.01	0.014
7	4.08	742	735	3-Penten-2-one	0.26±0.03b	0.65±0.05a	0.8±0.27a	0.66±0.06a	MS，RI	0.74	0.009
8	4.86	761	762	1-Pentanol	1.27±0.22b	2.88±0.17ab	3.66±1a	3.65±1.41a	MS，RI	0.77	0.031
9	4.98	764	762	Toluene	4±0.24b	5.25±0.6ab	8.38±2.71a	8.27±1.67a	MS，RI	0.95	0.024
10	6.02	789	793	Hexanal	500.94±74.48a	261.81±61.83b	551.2±50.28a	515.3±34.33a	MS, RI, Std	1.48	0.001
11	6.81	809	810	Acetic acid, butyl ester	5.4±0.43a	1.72±0.23b	2.53±0.73b	/	MS, RI, Std	1.20	0
12	8.27	845	821	3-Methylcyclopentanone	0.59±0.29a	0.46±0.11a	0.76±0.54a	0.89±0.25a	MS，RI	0.84	0.476
13	8.35	847	844	(*E*)-3-Hexen-1-ol	7.33±0.82b	16.93±2.81a	15.08±3.95a	5.52±4.6b	MS，RI	1.33	0.007
14	9.73	881	889	Styrene	0.39±0.16b	0.62±0.15b	0.79±0.2b	1.57±0.64a	MS，RI	1.27	0.016
15	10.36	897	897	Heptanal	71.17±3.21a	19.05±5.35d	35.27±1.37c	44.77±3.48b	MS, RI, Std	1.19	0
16	12.77	956	956	(*Z*)-2-Heptenal	0.22±0.03b	0.52±0.06ab	0.54±0.19ab	0.71±0.27a	MS，RI	0.93	0.04
17	12.88	959	959	Benzaldehyde	33.32±0.62a	22.92±2.3b	19.22±2b	20.84±2.25b	MS, RI, Std	0.89	0
18	13.37	971	970	1-Heptanol	5.92±1.48a	/	/	/	MS, RI, Std	0.94	0
19	14.05	988	986	1-Octen-3-ol	71.22±6.75a	40.02±11.11b	35.55±6.2b	41.85±4.36b	MS, RI, Std	0.82	0.002
20	14.13	990	990	6-Methyl-5-hepten-2-one	9.15±1.2a	6.62±1.32a	6.35±0.61a	7.15±1.81a	MS, RI, Std	0.68	0.107
21	14.17	991	993	2-Pentylfuran	5.48±0.69a	6.12±1.18a	8.73±3.6a	8.53±3.48a	MS，RI	0.82	0.364
22	14.76	1005	1004	Octanal	0.64±0.16a	1.16±0.13a	1.38±0.57a	0.81±0.55a	MS，RI	0.85	0.194

23	15.22	1017	1017	*α*-Terpinene	0.3±0.06a	0.55±0.1a	0.66±0.22a	0.65±0.46a	MS，RI	0.63	0.364
24	15.27	1018	1017	(*E*,-*E*)-2,4-Heptadienal	5.9±0.24a	5.09±0.85a	5.4±0.16a	5.78±0.58a	MS, RI, Std	0.85	0.295
25	15.58	1026	1025	*o*-Cymene	0.73±0.11a	1.88±0.41a	2.01±0.65a	2.49±1.24a	MS，RI	0.83	0.089
26	16.01	1036	1036	Benzyl alcohol	1.15±0.21a	1.24±0.16a	0.7±0.15a	1.09±0.29a	MS, RI, Std	1.02	0.056
27	16.21	1041	1041	Benzeneacetaldehyde	10.18±1.94b	29.4±5.17b	52.23±20.61a	16.18±8.46b	MS，RI	1.03	0.009
28	16.56	1050	1027	3-Carene	0.58±0.06b	2.51±0.41a	2.84±0.95a	2±1.23ab	MS，RI	0.77	0.037
29	16.98	1060	1060	*γ*-Terpinene	0.2±0.06b	0.54±0.04b	0.6±0.18b	1.32±0.45a	MS，RI	1.30	0.003
30	17.61	1076	1074	1-Octanol	1.43±0.27a	2.38±0.59a	4.29±1.12a	2.48±1.64a	MS，RI	0.83	0.054
31	18.3	1093	1092	2-Methoxyphenol	41.4±4a	57.2±9.61a	49.59±12.51a	57.96±18.6a	MS, RI, Std	0.76	0.377
32	18.47	1097	1091	(*E*,-*E*)-3,5-Octadien-2-one	0.59±0.12b	2.07±0.52a	3.13±0.89a	3.2±0.74a	MS，RI	0.90	0.003
33	19.09	1112	1112	Linalool	90.97±4.22a	124.31±18.86a	106.28±24.13a	126.83±40.03a	MS, RI, Std	0.80	0.333
34	19.16	1114	1112	1-Nonanal	27.66±10.74b	/	115.24±19.95a	/	MS, RI, Std	1.40	0
35	19.53	1123	1022	Phenylethyl alcohol	579.19±63.63a	256.86±25.33b	177.97±17.11c	288.75±36.77b	MS, RI, Std	0.88	0
36	20.36	1144	1141	3-Nonen-2-one	5.08±0.62a	3.08±0.52b	2.3±0.56b	3.31±0.69b	MS, RI, Std	0.79	0.003
37	21.61	1175	1173	trans-Linalool oxide	2.38±0.15b	5.96±0.19a	6.89±2.03a	2.49±2.11b	MS，RI	1.15	0.01
38	22.18	1189	1182	1-Nonanol	5.72±0.2a	3.52±0.41c	3.67±0.68c	4.52±0.12b	MS, RI, Std	1.02	0.001
39	22.51	1197	1197	Methyl salicylate	3.45±0.25b	3.87±0.21b	4.5±1.13b	5.95±0.83a	MS, RI, Std	1.29	0.013
40	22.78	1203	1202	Decanal	0.2±0.03a	0.49±0.09a	0.55±0.25a	0.34±0.08a	MS，RI	0.87	0.054
41	23.22	1214	1217	*β*-Cyclocitral	0.95±0.08b	1.7±0.09b	3.44±1.06a	2.06±0.65b	MS，RI	0.90	0.008
42	23.52	1222	1223	(*E*,-*E*)-2,4-Nonadienal	7.04±0.11a	6.63±0.4a	6.98±0.5a	6.86±0.15a	MS, RI, Std	0.73	0.488
43	24.05	1235	1235	Nerol	902.07±51.74a	918.05±54.14a	0±0b	1026.35±192.33a	MS, RI, Std	1.36	0
44	24.78	1253	1252	Geraniol	46.73±2.6a	51.38±3.59a	43.23±15.39a	53.11±9.89a	MS, RI, Std	0.72	0.587
45	25.3	1266	1247	Citral	88.24±15.91a	/	/	/	MS, RI, Std	0.95	0
46	25.7	1276	1290	Indole	0.71±0a	0.7±0.01a	0.71±0.03a	0.69±0a	MS, RI, Std	0.76	0.505
47	25.71	1276	n.f	Dihydroactinidiolide	0.68±0.18a	0.1±0.01b	0.17±0.06b	0.49±0.07a	MS, RI, Std	1.17	0

48	26.82	1318	1319	Methyl geranate	0.24±0.04b	0.88±0.05a	0.8±0.22a	0.22±0.16b	MS，RI	1.35	0
49	27.31	1332	1331	(*E,-E*)-2,4-Decadienal	4.01±0.02a	3.98±0.04a	4±0.02a	3.96±0.04a	MS, RI, Std	0.91	0.246
50	29.2	1384	n.f	(*Z*)-Hexanoic acid, 3-hexenyl ester	0.81±0.07a	0.61±0.05b	0.67±0.06b	0.67±0.05b	MS, RI, Std	0.95	0.017
51	29.7	1397	1398	*cis*-Jasmone	12.55±0.82a	10.26±2ab	5.69±1.33c	8.57±1.79bc	MS, RI, Std	0.89	0.004
52	31.4	1444	1444	Geranyl acetone	3.14±0.24a	1.7±0.29b	1.29±0.43b	1.61±0.26b	MS, RI, Std	0.87	0
53	32.3	1468	1476	*β*-Ionone	0.97±0.12a	1.15±0.34a	0.41±0.24b	0.75±0.12ab	MS, RI, Std	1.19	0.02
54	32.52	1474	1467	*α*-Muurolene	0.23±0.09b	0.41±0.03b	0.85±0.28a	0.82±0.14a	MS，RI	1.02	0.004
55	33.12	1491	1481	(-)-*β*-Cadinene	2.5±0.57c	3.79±0.34bc	7.08±2.27a	5.61±1.73ab	MS，RI	0.86	0.022
56	33.22	1493	1496	*cis*-Calamenene	4.28±0.69c	7.28±0.94bc	18.11±6.3a	12.87±3.66ab	MS，RI	0.94	0.008
57	33.55	1502	1503	Cubenene	0.42±0.13b	0.61±0.03b	1.12±0.38a	0.52±0.29b	MS，RI	0.97	0.037
58	33.81	1509	1520	*α*-Calacorene	0.49±0.11b	1.04±0.16b	2.1±0.8a	0.7±0.4b	MS，RI	1.02	0.011
59	33.85	1511	1514	Nerolidol	1.8±0.02a	1.69±0.03b	1.73±0.02b	1.7±0.01b	MS, RI, Std	1.04	0.001
60	34.42	1526	1542	*β*-Calacorene	0.06±0.01b	0.12±0.01b	0.26±0.1a	0.18±0.07ab	MS，RI	0.85	0.021

Note: FGT represents flat green tea; PGT represents phoenix green tea; CGT represents curled green tea; SGT represents straight green tea.

All analyses were performed in triplicate, and the results were expressed as mean ± SD (n = 3).

RT[a] represents retention time; RI[b] represents the linear retention indices calculated from a series of *n*-alkanes (C7-C40); RI[c] represents retention indices referred to the literature value with HP-5ms column or equivalent chromatographic column [NIST Chemistry WebBook (http://webbook.nist.gov/chemistry/) and http://www.flavornet.org/flavornet.html/]. ID represents identification method. MS represents identification based on the NIST 11 database; Std represents standard certification. “n.f.” represents data not found in the literature; VIP represents variable importance in the projection.

Table S5 The OAVs of volatile compounds in tea samples with four distinct shaping techniques.

No.	Compounds	OT (μg/L)	OAVs			
			PGT	CGT	FGT	SGT
1	3-Methylbutanal	0.5 [A]	2.51	14.88	5.33	10.71
2	2-Methylbutanal	1.5 [A]	0.73	2.24	1.97	3.48
3	1-Penten-3-ol	400 [A]	0	0.01	0.01	0.01
4	*cis*-2-Penten-1-ol	720 [B]	0.25	0	0.4	0.3
5	Pentanal	12 [A]	0.18	0.38	0.47	0.24
6	2-Ethylfuran	8000 [D]	0	0	0	0
7	3-Penten-2-one	n.f.	/	/	/	/
8	1-Pentanol	5000 [A]	0	0	0	0
9	Toluene	140 [A]	0.03	0.04	0.06	0.06
10	Hexanal	4.5 [C]	111.32	58.18	122.49	114.51
11	Acetic acid, butyl ester	n.f.	/	/	/	/
12	3-Methylcyclopentanone	n.f.	/	/	/	/
13	(*E*)-3-Hexen-1-ol	110 [C]	0.07	0.15	0.14	0.05
14	Styrene	n.f.	/	/	/	/
15	Heptanal	0.9 [A]	79.08	21.17	39.19	49.75
16	(*Z*)-2-Heptenal	0.15 [A]	1.43	3.47	3.63	4.71
17	Benzaldehyde	50 [D]	0.67	0.46	0.38	0.42
18	1-Heptanol	400 [A]	0.01	0	0	0
19	1-Octen-3-ol	1 [D]	71.22	40.02	35.55	41.85
20	6-Methyl-5-hepten-2-one	0.16 [A]	57.22	41.36	39.68	44.67
21	2-Pentylfuran	n.f.	/	/	/	/
22	Octanal	7 [D]	0.09	0.17	0.2	0.12

23	*α*-Terpinene	n.f.	/	/	/	/
24	(*E*,-*E*)-2,4-Heptadienal	10000 B	0	0	0	0
25	*o*-Cymene	11.4 B	0.06	0.16	0.18	0.22
26	Benzyl alcohol	100 A	0.01	0.01	0.01	0.01
27	Benzeneacetaldehyde	1.2 A	8.49	24.5	43.52	13.48
28	3-Carene	4000 A	0	0	0	0
29	*γ*-Terpinene	n.f.	/	/	/	/
30	1-Octanol	3 A	0.48	0.79	1.43	0.83
31	2-Methoxyphenol	0.84 A	49.28	68.1	59.04	69
32	(*E*,-*E*)-3,5-Octadien-2-one	n.f.	/	/	/	/
33	Linalool	0.6 D	151.61	207.18	177.14	211.39
34	1-Nonanal	n.f.	/	/	/	/
35	Phenylethyl alcohol	0.35 A	1654.82	733.87	508.48	825
36	3-Nonen-2-one	n.f.	/	/	/	/
37	trans-Linalool oxide	3000 A	0	0	0	0
38	1-Nonanol	45.5 C	0.13	0.08	0.08	0.1
39	Methyl salicylate	40 D	0.09	0.1	0.11	0.15
40	Decanal	2.6 A	0.08	0.19	0.21	0.13
41	*β*-Cyclocitral	3 A	0.32	0.57	1.15	0.69
42	(*E*,-*E*)-2,4-Nonadienal	0.06 A	117.4	110.52	116.35	114.4
43	Nerol	290 A	3.11	3.17	0	3.54
44	Geraniol	7.5 B	6.23	6.85	5.76	7.08
45	Citral	40 C	2.21	0	0	00
46	Indole	11 A	0.06	0.06	0.06	0.06
47	Dihydroactinidiolide	500 A	0	0	0	0
48	Methyl geranate	n.f.	/	/	/	/

49	(*E,-E*)-2,4-Decadienal	0.027 [A]	148.56	147.26	148.14	146.76
50	(*Z*)-Hexanoic acid, 3-hexenyl ester	n.f.	/	/	/	/
51	*cis*-Jasmone	7 [C]	1.79	1.47	0.81	1.22
52	Geranyl acetone	60 [A]	0.05	0.03	0.02	0.03
53	*β*-Ionone	0.01 [B]	96.84	115.05	41.45	75.35
54	*α*-Muurolene	n.f.	/	/	/	/
55	(-)-*β*-Cadinene	n.f.	/	/	/	/
56	*cis*-Calamenene	n.f.	/	/	/	/
57	Cubenene	n.f.	/	/	/	/
58	*α*-Calacorene	n.f.	/	/	/	/
59	Nerolidol	10 [C]	0.18	0.17	0.17	0.17
60	*β*-Calacorene	nf	/	/	/	/

Note: FGT represents flat green tea. PGT represents phoenix green tea. CGT represents curled green tea. SGT represents straight green tea.

OT represents the threshold of the volatile compound in water. [A] (Zhai, Zhang, Granvogl, Ho, & Wan, 2022); [B] (Van Gemert, 2011); [C] (Xie et al., 2023); [D] (Guo, Ho, Wan, Zhu, Liu, & Wen, 2021)

[A] Zhai, X., Zhang, L., Granvogl, M., Ho, C.-T., & Wan, X. (2022). Flavor of tea (Camellia sinensis): A review on odorants and analytical techniques. Comprehensive Reviews in Food Science and Food Safety, 21(5), 3867-3909. https://doi.org/10.1111/1541-4337.12999.

[B] Van Gemert, L.J. ODOUR THRESHOLDS—Compilations of Odour Threshold Values in Air, Water and Other Media; Oliemans Punter & Partners BV: Zeist, The Netherlands, 2011.

[C] Xie, J., Wang, L., Deng, Y., Yuan, H., Zhu, J., Jiang, Y., & Yang, Y. (2023). Characterization of the key odorants in floral aroma green tea based on GC-E-Nose, GC-IMS, GC-MS and aroma recombination and investigation of the dynamic changes and aroma formation during processing. Food Chemistry, 427, 136641. https://doi.org/https://doi.org/10.1016/j.foodchem.2023.136641.

[D] Guo, X., Ho, C.-T., Wan, X., Zhu, H., Liu, Q., & Wen, Z. (2021). Changes of volatile compounds and odor profiles in Wuyi rock tea during processing. Food Chemistry, 341, 128230. https://doi.org/https://doi.org/10.1016/j.foodchem.2020.128230.

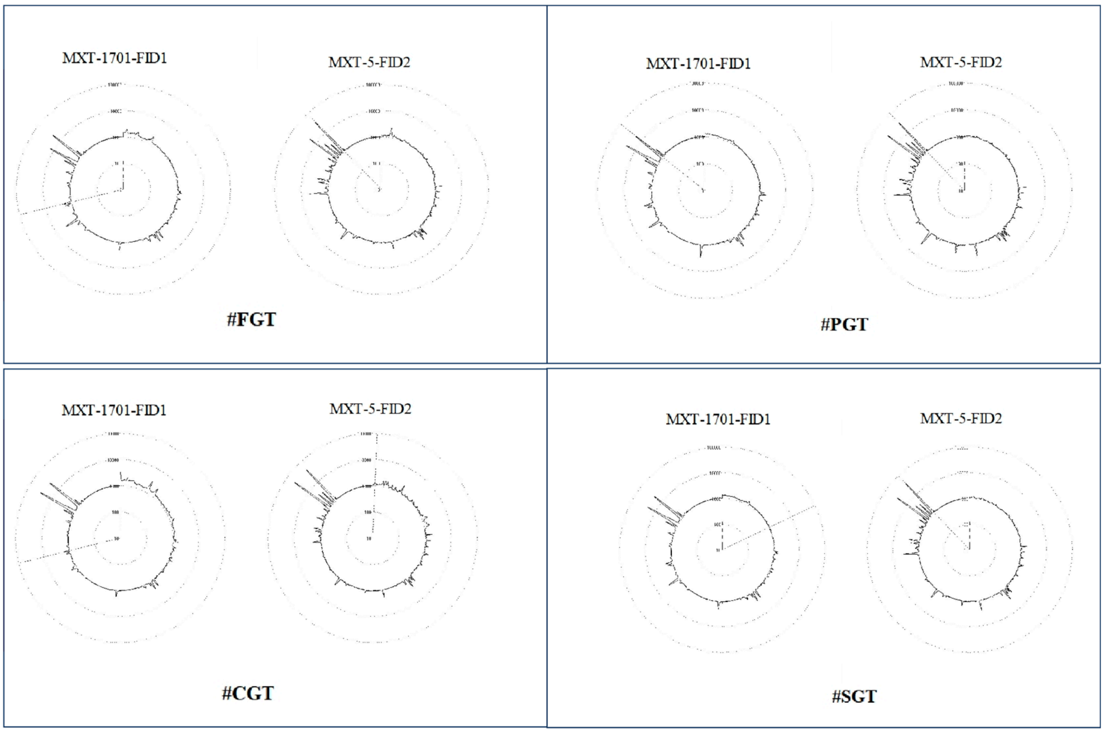

Figure S1 The volatile fingerprints of green tea with different shaping techniques obtained from the MXT-5 and MXT-1701 columns in parallel.

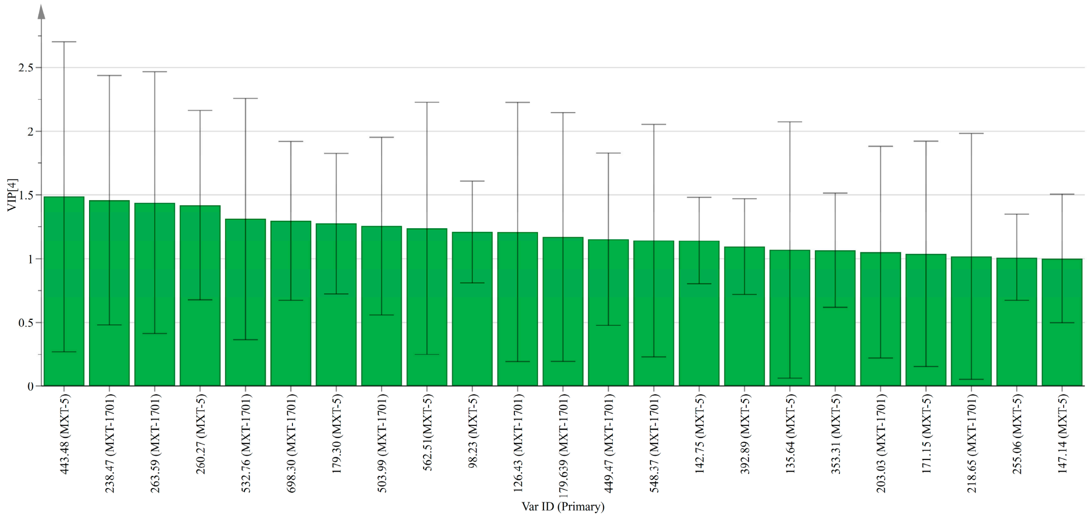

Figure S2 A total of 23 variables with VIP greater than 1 obtained from GC-E-Nose.
